# Construction of a highly saturated genetic map and identification of quantitative trait loci for leaf traits in jujube

**DOI:** 10.3389/fpls.2022.1001850

**Published:** 2022-10-06

**Authors:** Fenfen Yan, Yujia Luo, Jingkai Bao, Yiling Pan, Jiurui Wang, Cuiyun Wu, Mengjun Liu

**Affiliations:** ^1^ College of Horticulture and Forestry, Tarim University/The National and Local Joint Engineering Laboratory of High Efficiency and Superior-Quality Cultivation and Fruit Deep Processing Technology of Characteristic Fruit Trees in Southern Xinjiang, Alar, China; ^2^ Xinjiang Production and Construction Crops Key Laboratory of Protection and Utilization of Biological Resources in Tarim Basin, Tarim University, Alar, China; ^3^ College of Forestry, Hebei Agricultural University, Baoding, China; ^4^ College of Horticulture, Hebei Agricultural University, Baoding, China

**Keywords:** Genetic map, *Ziziphus jujuba* mill, Whole-genome resequencing (WGR), Leaf traits, qtl

## Abstract

Chinese jujube (*Ziziphus jujuba* Mill.), a member of the genus *Ziziphus*, which comes under the family Rhamnaceae, is the most important species in terms of its economic, ecological, and social benefits. To dissect the loci associated with important phenotypical traits and analyze their genetic and genomic information in jujube, a whole-genome resequencing (WGR) based highly saturated genetic map was constructed using an F1 hybrid population of 140 progeny individuals derived from the cross of ‘JMS2’ × ‘Jiaocheng 5’. The average sequencing depth of the parents was 14.09× and that of the progeny was 2.62×, and the average comparison efficiency between the sample and the reference genome was 97.09%. Three sets of genetic maps were constructed for a female parent, a male parent, and integrated. A total of 8,684 markers, including 8,158 SNP and 526 InDel markers, were evenly distributed across all 12 linkage groups (LGs) in the integrated map, spanning 1,713.22 cM with an average marker interval of 0.2 cM. In terms of marker number and density, this is the most saturated genetic map of jujube to date, nearly doubling that of the best ones previously reported. Based on this genetic map and phenotype data from 2019 to 2021, 31 leaf trait QTLs were identified in the linkage groups (LG1, 15; LG3, 1; LG5, 8; LG7, 4; LG8, 1, and LG11, 2), including 17 major QTLs. There were 4, 8, 14, and 5 QTLs that contributed to leaf length, leaf width, leaf shape index, and leaf area, respectively. Six QTLs clusters were detected on LG1 (8.05 cM–9.52 cM; 13.12 cM–13.99 cM; 123.84 cM–126.09 cM), LG5 (50.58 cM–50.86 cM; 80.10 cM–81.76 cM) and LG11 (35.98 cM–48.62 cM). Eight candidate genes were identified within the QTLs cluster regions. Annotation information showed that 4 genes (LOC107418196, LOC107418241, LOC107417968, and LOC112492570) in these QTLs are related to cell division and cell wall integrity. This research will provide a valuable tool for further QTL analysis, candidate gene identification, map-based gene cloning, comparative mapping, and marker-assisted selection (MAS) in jujube.

## Introduction

Jujube (*Ziziphus jujuba* Mill.) is the most important species of the family Rhamnaceae and an increasingly popular fruit tree crop in the world in terms of its economic, ecological, and social benefits (Liu et al., 2020). Jujube is drought and salinity tolerant, making it suitable for cultivation in arid and semi-arid marginal regions of the world. Its fruit is a well-known nourishing fruit and traditional herbal medicine, as well as an excellent source of vitamin C and sugar (Liu and Wang, 2019). It is currently one of the most important cultivated fruit species, with a growing area of 2 million hectares, producing about 8 million tons annually, and is also the primary source of income for 20 million farmers in China (Huang et al., 2016; Liu et al., 2020).

Marker-assisted selection (MAS) has been proven to be an efficient approach for quickening the breeding of fruit trees. Furthermore, MAS is generally based on high-density genetic linkage maps to develop molecular markers for high-throughput selection of superior traits (Zhu et al., 2018; Gao et al., 2020). The most important agronomic traits of fruit trees are quantitative traits, such as organ size, yield, fruit quality, stress resistance, and so on, and their phenotypic traits have a continuous normal distribution in offspring (Liu et al., 2016; Wang et al., 2019). Quantitative trait loci (QTL) analysis based on a genetic linkage map is an important method in studying molecular assisted breeding and functional genomics (Wu et al., 2014; Xu et al., 2015; Ma et al., 2020). Consequently, a high-density genetic map is needed to facilitate various genetic studies and meet the increasing demand for cultivar improvement of jujube.

Some progress has been made in the development of molecular markers and the construction of molecular linkage maps in jujube. Before the jujube genome was sequenced in 2014, a few genetic maps were constructed in jujube by using different types of markers, such as amplified fragment length polymorphisms (AFLPs), random amplified polymorphic DNAs (RAPDs), and simple sequence repeats (SSRs). These maps were limited by low marker density and had some inconsistencies in LG number, which has prevented the fine mapping of target traits (Lu, 2003; Shen, 2005; Qi, 2006; Xu, 2012). Since the jujube genome sequence was released (Liu et al., 2014; Huang et al., 2016), the development of massive SNP markers and the construction of a high-density genetic map in jujube became possible. Until now, four saturated reference maps using a simplified genome sequencing technique have been published for jujube derived from different F1 progeny from 2014 to 2019 (Zhao et al., 2014; Zhang, 2016; Zhang et al., 2016; Wang et al., 2019). The above-mentioned simplified genome sequencing technologies such as GBS and RAD-seq are relatively inexpensive, but parts of incomplete data might be produced (Sun et al., 2015; Zhu et al., 2018). However, it is still far behind many other fruits in the genetic map density, the functional genetic mapping and marker-assisted selection in jujube. Whole genome resequencing (WGS) is a technique for developing molecular markers based on reference genome information that has higher efficiency and accuracy compared to other methods (Li et al., 2018). In addition to a large number of SNPs, a large number of InDel information can also be generated by WGS, which is present in co-dominance and widely distributed in the genome. QTL analysis mostly relies on polymorphisms between the parents of mapping populations and different hybrid progeny, which show different marker genotypes or linkage relationships. Thus, there is an urgent need to develop a highly saturated genetic linkage map in jujube for marker-assisted breeding and genome investigations.

The leaf is an important organ that undergoes photosynthesis in the plant and serves as the foundation for a high and stable yield. The size and shape of the leaves can directly affect the plant productivity and reflect the characteristics of the cultivars (Wang et al., 2019a). The inheritance of leaf size traits has been proven to be quantitative, and 27 QTLs for leaf traits were detected based on the genetic map (Wang et al., 2019). Among them, QTLs linked to leaf length were found on LG1 and LG11 based on two maps (Shen, 2005; Wang et al., 2019), but most QTLs linked to leaf traits always have low rates for the explanation of variance. Thus, mapping of genes controlling leaf traits and the development of applicable markers will be of great significance for molecular-assisted breeding in jujube.

In this study, we constructed a highly saturated genetic linkage map of jujube by using WGS technology with a F1 population of 140 progeny individuals derived from a cross between ‘JMS2’ (a male sterile cultivar) and ‘Jiaocheng 5’ (a cultivar with high resistance to phytoplasma disease and big fruit). Then, QTL analysis was performed to identify the genomic regions associated with leaf traits. These results could provide useful information for marker-assisted breeding and increase understanding of the genetic control of leaf traits in jujube.

## Materials and methods

### Plant material and DNA extraction

The F1 jujube hybrids of 140 progeny individuals derived from a cross between ‘JMS2’ and ‘Jiaocheng 5’ were generated in 2016. The female parent is a male sterile table cultivar, with a medium-sized oval fruit shape, excellent quality, high yield, and good adaptability. The male parent is an elite strain of ‘Junzao’ with extremely high resistance to phytoplasma disease and a long cylinder shape with big-size fruit. Hybrid seeds were sown in a greenhouse in 2016. A total of 178 hybrids were randomly harvested, of which 140 of them and two parents were used as the mapping population. All plant materials were grafted on the rootstock of 7-year-old ‘Junzao jujube’ in Aral, Xinjiang, China (40°59’N, 81°28’E) in April 2018. Each hybrid seedling was grafted with three main branches as three biological replicates. It grew in soil sand and a desert climate in the orchard. Common ways of orchard management were applied.

Investigations of the progeny of individuals were performed from 2019 to 2021. Healthy young leaves (second or third leaf from the apex of the bearing shoot, less than 1 cm^2^) were harvested from both parents and each individual progeny plant (F1 generation). The samples were stored in an −80°C refrigerator. Genomic DNA was extracted using the improved CTAB method (Xin and Chen, 2012). DNA concentration and quality were evaluated using the NanoDrop 2000 spectrophotometer (Thermo Fisher Scientific, Waltham, MA, USA). Finally, the concentration and volume of each DNA sample were 500 ng·μl^−1^ and 50 μl, respectively. The quality of DNA was electrophoresed on a 1% agarose gel. The DNA was diluted to a final concentration of 2.5 ng·μl^−1^ for the next steps.

### Sequencing library construction

After checking the quality of DNA, the DNA sequence was segmented into random 200–500 bp fragments by ultrasonic. The sequencing libraries were constructed using terminal repair, followed by the addition of a 3’ end with A and a sequencing linker. The samples were then purified and amplified by PCR. After quality inspection, the qualified libraries were sequenced using the Illumina HiSeqTM platform using the Illumina PE150-sequencing strategy, and the total sequencing read length was 300 bp. The sequencing library construction was completed by the Shanghai Magi company.

### SNP and InDel identification and genotyping

Raw reads from the Illumina HiseqTM were filtered to obtain high-quality reads (clean reads), then the clean reads were re-mapped to the reference genome: the ‘Dongzao’ genome (https://www.ncbi.nlm.nih.gov/genome/?term=Ziziphus+jujuba) was used as the reference genome to get the sequence position (BWA files) by BWA software (Li and Richard, 2009). The best practice process of GATK (McKenna et al., 2010) was used for base recalibration, variant calling, and strictly filtering the SNPs and Small InDel to correct the BAM files. To guarantee the quality of the genetic map, SNPs and Small InDel were filtered: (1) The depth of markers from the parental line was more than 10×, and the progeny depth was more than 2×. (2) The marker confirms the characteristics of the F1 population, at least one parent was selected as heterozygous typing. (3) The progeny separation ratio confirms to the Mendelian separation ratio (p >0.05). (4) The absence rate of progeny typing was less than 23%.

Gene genotyping of the progenies was based on the parental genotype. The parental line with high sequencing depth could guarantee that the genotyping of the progenies is right. The average sequencing depth was 13-fold in the parents and 3.32-fold in the progenies. Variants filtered by quality as described above were genotyped in accordance with their heterozygous parents into eight segregation types. After filtering those with no polymorphisms between parents or partial separation based on a P-value <0.01, markers with homozygous parents were used to construct a genetic linkage map for the F1 generation.

### Genetic map construction and evaluation

Genetic marker data were scored in accordance with the criteria of JoinMap v.5.0. A logarithm of the odds (LOD) score of 4–20 was set to distinguish linkage groups (Danny et al., 2010). Regression mapping was used as the mapping algorithm, and the genetic distances were calculated on the basis of Kosambi’s mapping function (Kosambi, 2011).

Based on the basic principle of the genetic map, the genetic map was constructed by a pseudo-test cross in a linkage group. Due to the F1 integrating the restructuring events of the female parent and male parent, respectively, in meiosis, both the female genetic map and the male genetic map were constructed separately, then the integrated maps were constructed by integrating all recombination events of common molecular markers between the female and male maps. The whole analysis process was completed by Crosslink analysis. All of the sequences of the bin markers that were used to construct the linkage map were aligned to the physical sequences of the reference genome. Collinearity between genetic and physical positions was determined by plotting genetic marker positions against their physical positions, and the BLAST program was used to confirm their physical positions in the genome (Li et al., 2018). The Spearman correlation coefficients were calculated to assess the collinearity between the genetic and physical maps.

### Evaluation of phenotypes

The leaf traits of 140 hybrids and their parents were investigated from September 2019 to September 2021. Thirty leaves were picked from the middle of the bearing shoots on each tree, which were potted in an ice box to maintain freshness. Four major leaf traits, including leaf length, leaf width, leaf area, and leaf shape index, were scanned and analyzed by the LA-S plant image analyzer system. All data were analyzed using SPSS 26.0 to calculate the mean, standard deviation, kurtosis, and skewness.

### QTL analysis tables

QTL locations were conducted with Internal Mapping and R/QTL software (Broman et al., 2003) was used for QTL analysis. The permutation test was used to determine the limit of detection (LOD). The threshold of the LOD score for significance (*p* = 0.05) was determined using 1,000 permutations. The calculation of the percentage of phenotypic variance explained by each QTL was based on the population variance found within the segregation population. A LOD threshold of 3.0 was set to identify leaf QTLs at the 95% confidence level, except a LOD threshold of 2.7 was set to identify QTLs of leaf width in 2019. Ranges above the LOD threshold of 3.0 were identified as QTL intervals, and those higher than 3.5 were considered major loci.

Based on the positions of the flanking markers, all of the genes within the confidence interval were identified as candidates. The candidate genes in the target locus region were annotated from the Jujube reference genome. Annotations from the GO and KEGG databases were used to categorize the candidate genes. Candidate regions were named according to the naming system rules (Mccouch, et al. 1997) as follows: q + trait abbreviation + year abbreviation + locus number. The loci for the same trait across different generations and environments were considered common loci when their confidence intervals overlapped.

## Results

### Quality evaluation of sequencing data

The sequencing library with an insert fragment size of about 400 bp was constructed by the Illumina Novaseq 6000 database building sequencing platform. After filtering low-quality bases, a total of 203.02-G clean reads were obtained for 142 sample individuals, with an average of 1.43-G clean data for each sample. The Q30 ratio of both parents was 93.48%, and that of F1 progeny ranged from 90.26% to 93.56%, with an average of 92.03%, indicating the high quality of sequencing data (**Table 1**). The GC content of the parents was 35%, while that of the hybrid population ranged from 33.96% to 36.48%, with an average of 34.84%.

**Table 1:** Quality evaluation of sequencing data in 140 hybrid progeny and their parent.

Sample		Insert Size(Bp)	Raw Bases (Gb)	Clean Reads	Clean Bases(Gb)	Clean GC(%)	Q30(%)	Genome Coverage (1×) (%)	Genome Coverage (5×) (%)
Parents	Male	320.50	6.76	45,149,838	6.76	34.58	93.48	87.99	73.55
	Female	323.30	6.62	44,208,686	6,62	35.50	93.48	88.57	75.96
Progeny	Maximum	444.60	1.84	12,308,564	1,84	36.48	93.57	79.13	23.91
	Minimum	294.00	0.93	6,249,186	0.93	33.96	90.27	63.77	6.04
	Average	330.93	1.35	9,045,778	1.35	34.84	92.04	73.12	13.01

Clean reads were compared to the published reference jujube genome sequences (Liu et al., 2014) using BWA software. The mapping ratios of the two parents, ‘JMS2’ and ‘Jiaocheng 5,’ were 96.98% and 97.17%, respectively, and the average comparison efficiency between the sample and the reference genome was 97.09%. The average sequencing depth of the parents and progeny was 14.09×, and that of the progeny was 2.62×, respectively. The average genome coverage (1×) was 73.33% (at least one base coverage).

### SNP and InDel discovery and genotyping

The SNP and InDel markers were detected by GATK. A total of 4,588,795 SNPs and 1,078,886 InDel markers were obtained. Then, these markers were filtered, and screened by the screening criteria. At least 14,167 markers were obtained conforming to the construction of the genetic map, which could be divided into eight segregation patterns as follows: aa×bb, ab×cc, ab×cd, cc×ab, ef×eg, hk×hk, lm×ll, and nn×np (**Table 2**). Since this study was conducted in the F1 population, segregation patterns except aa× bb type were selected as effective markers for population composition. At last, the effective markers that could be used for genetic map construction accounted for 89.48%.

**Table 2:** Genotyping and number of markers.

Marker Tape	paternal genotype	maternal genotype	SNP Marker	InDel Marker
aa×bb	aa	bb	349240	82619
ab×cc	ab	cc	2085	5895
ab×cd	ab	cd	13	1756
cc×ab	cc	ab	2628	6093
ef×eg	ef	eg	12417	19948
hk×hk	hk	hk	821320	119575
lm×ll	lm	ll	1102495	201296
nn×np	nn	np	1172489	203743
Total	–	–	3462687	640925

### Genetic linkage map construction

Linkage clustering was carried out according to the LOD value between genomic information and markers, and an LOD value between 4 and 20 was selected in this clustering scheme. Meanwhile, the number of linkage groups (LG) was kept consistent with the number of jujube chromosomes (12). Finally, the above 14,167 markers were divided into 12 linkage groups after correcting and filtering, and a total of 8,684 markers were found in the linkage groups (**Table 3**). Both SNP and InDel markers were distributed on all 12 chromosomes. Among them, the LG5 contained 1,792 markers (1,684 SNP and 108 InDel), which has the largest number of markers. LG2 and LG1 were not far behind, containing 1,135 and 1,019 markers, respectively, while LG12 had the fewest markers (233).

**Table 3:** Distribution of markers of integrated map in 12 linkage groups.

LG	Marker number	SNP number	InDel number
1	1019	964	55
2	1135	1068	67
3	642	599	43
4	656	608	48
5	1792	1684	108
6	429	406	23
7	839	785	54
8	461	441	20
9	527	496	31
10	563	530	33
11	388	365	23
12	233	212	21

Based on the basic principle of genetic mapping, the genetic map was constructed by pseudo-testcross for the F1 group. Due to the F1 group integrating experienced the recombination event of female parent and male parent at the time of the separation, the construction of the F1 population map should build maternal and paternal separate genetic map firstly, then all the recombination events should be integrated using common molecular markers between the female and male maps, and the integrated map was obtained by cross-link analysis. Finally, three sets of maps were obtained, including female, male, and integrated. The specific statistical results are also shown in **Table 3**.

The female map contained 5,097 markers with a total length of 1,842.47 cM and the average distance between markers was about 0.36 cM (**Table 4**). Among the 12 LGs, LG5 contained the most markers (1,163) with a genetic distance of 160.78 cM and an average marker interval of 0.14 cM, whereas LG12 contained the fewest (123) markers, spanning 169.82 cM, with an average marker interval of 1.39 cM. The largest gap was detected on LG2 with a distance of 42.46 cM, and the smallest gap was detected on LG4 with a distance of 6.31 cM. The distance between adjacent markers was more than 5 cM (‘Gap >5 cM’), which ranged from 2 (LG3, LG4, LG5, and LG10) to 8 (LG11).

**Table 4:** Basic characteristics of Jujube linkage groups bout Female map,Male map and Integrated map.

LG	Total marker			Total distance			Average distance			Gap > 5cM			Max gap		
	Female map	Male map	Integrated map	Female map	Male map	Integrated map	Female map	Male map	Integrated map	Female map	Male map	Integrated map	Female map	Male map	Integrated map
1	556	742	1019	152.41	157.24	150.40	0.27	0.21	0.15	4	2	0	6.32	6.68	3.47
2	370	902	1135	149.41	151.04	128.78	0.4	0.17	0.11	5	1	1	42.46	9.04	8.04
3	457	420	642	126.31	134.91	154.05	0.28	0.32	0.24	2	1	2	11.11	18.08	9.13
4	397	453	656	154.19	164.40	168.09	0.39	0.36	0.26	2	1	0	6.31	6.31	4.53
5	1163	1472	1792	160.78	156.08	120.03	0.14	0.11	0.07	2	0	1	18.23	2.80	8.14
6	319	262	429	160.91	128.18	146.93	0.51	0.49	0.34	4	3	2	9.61	10.19	6.17
7	477	554	839	169.72	163.23	129.27	0.36	0.3	0.15	5	3	1	33.39	8.88	7.42
8	268	346	461	143.20	149.29	162.53	0.54	0.43	0.35	7	2	0	11.24	9.99	4.69
9	334	340	527	160.84	138.23	123.33	0.48	0.41	0.23	6	1	5	14.42	6.43	7.90
10	384	362	563	128.89	146.15	166.53	0.34	0.4	0.3	2	5	2	6.73	9.68	6.35
11	249	259	388	166.00	163.89	141.50	0.67	0.64	0.37	8	10	2	11.10	8.93	5.74
12	123	188	233	169.82	160.98	121.78	1.39	0.86	0.52	7	7	1	10.45	17.68	5.10
total	5097	6300	8684	1842.47	1813.62	1713.22	0.36	0.29	0.2	54	36	17	42.46	18.08	9.13

The map of the male parent contained 6,300 markers spanning 1,813.62 cM with an average distance of about 0.29 cM between markers (**Table 4**). LG5 (1,472) and LG12 (188) contained the most and the least markers, with the shortest (0.11 cM) and the longest (0.86 cM) being the average distance between markers, respectively. The length of each LG ranged from 128.18 cM for LG6 to 164.40 cM for LG4. The largest gap (18.08 cM) was detected on LG3 and the smallest gap (2.80 cM) was detected on LG5. There were 36 ‘Gap > 5 cM’ detected in this map, including most of 10 on LG11 and at least 0 on LG5.

**Figure 1:**
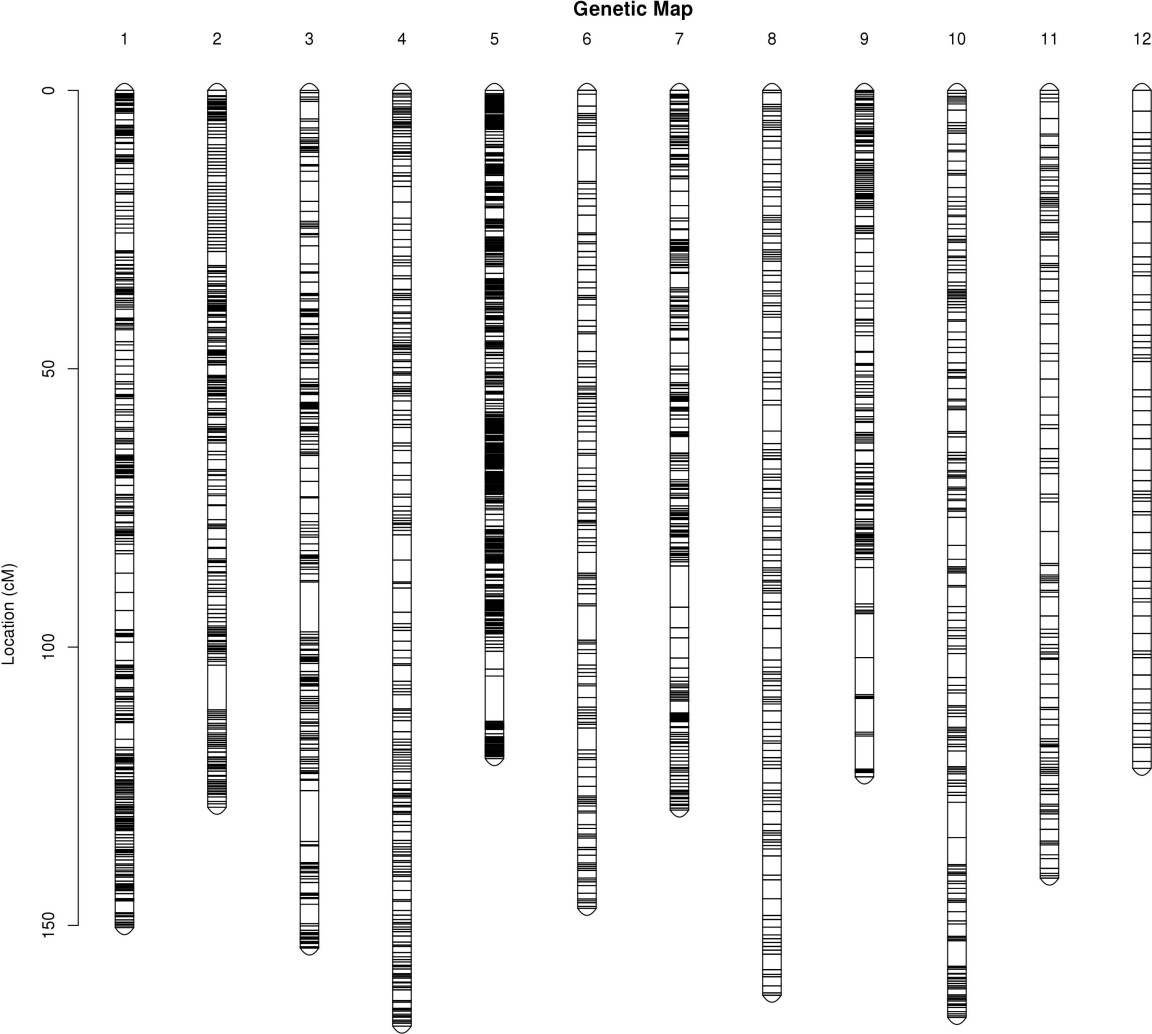
Genetic linkage groups of integrated map.

The integrated map contained a set of 8,684 markers spanning 1,713.22 cM, with an average inter-marker distance of 0.2 cM (**Table 4**; **Figure 1**). The genetic length of LGs ranged from 120.03 cM (LG5) to 168.09 cM (LG4), with an average length of 142.77 cM. LG5 contained the most markers (1,792) spanning 120.03 cM with an average genetic interval of 0.07 cM, whereas LG12 spanned 121.78 cM and contained the fewest markers (233) with an average genetic interval of 0.52 cM. Similarly, LG2 contained 1,135 markers with an average genetic interval of 0.11 cM, and LG1 contained 1,019 markers with an average genetic interval of 0.15 cM. Only 17 of ‘Gap >5’ were observed in nine LGs, among which LG9 contained 5 with a max gap of 7.9 cM and LG3 had a max gap of 9.13 cM. There was no’Gap >5’ in LG1, LG4 and LG8, which indicated the markers were well-distributed on the genome.

### Collinearity of the genetic and physical maps

**Figure 2:**
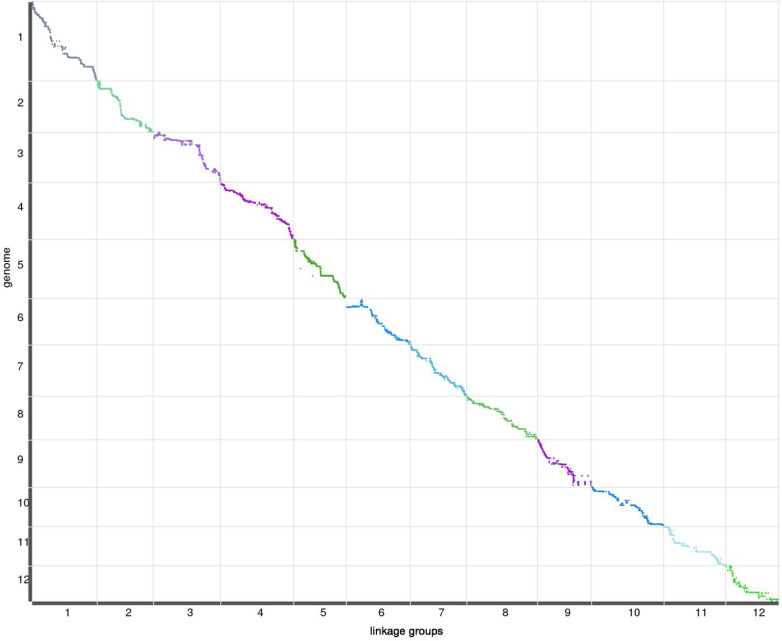
Relationship between genetic and physical positions with each chromosome.In each plot, genetic distance is on the x-axis, and physical distance is on the y-axis.

The purpose of the genetic map is to analyze multi-point recombination; the closer the distance between markers, the lower the recombination rate. To evaluate the quality of the genetic map, heat maps of the 12 LGs were generated separately based on pair-wise recombination values for the 8,684 recombination bin markers. The linkage relation heat map illustrated the relationship between recombination markers on one chromosome, which generally indicated that the construction of the present genetic map was accurate. The correlation between genetic and physical positions on a linkage map defines its quality. To compare genetic and physical maps, we investigated the locations of all SNP and InDel markers on the reference genome (**Figure 2**). A high degree of collinearity was observed between genetic and physical distances in the 12 LGs, and the absolute values of Spearman correlation coefficients were all greater than 98%, except LG6, meaning the value of 99%. All consecutive curves generated from the 12 LGs indicated that they have high genetic collinearity with the physical positions of each chromosome, and markers covered 12 chromosomes were positioned accurately, which was sufficient to cover the jujube genome. Our high-density genetic map overlaps 97.09% of the physical map of Jujube.

### Characterization of leaf traits in the mapping population

**Figure 3:**
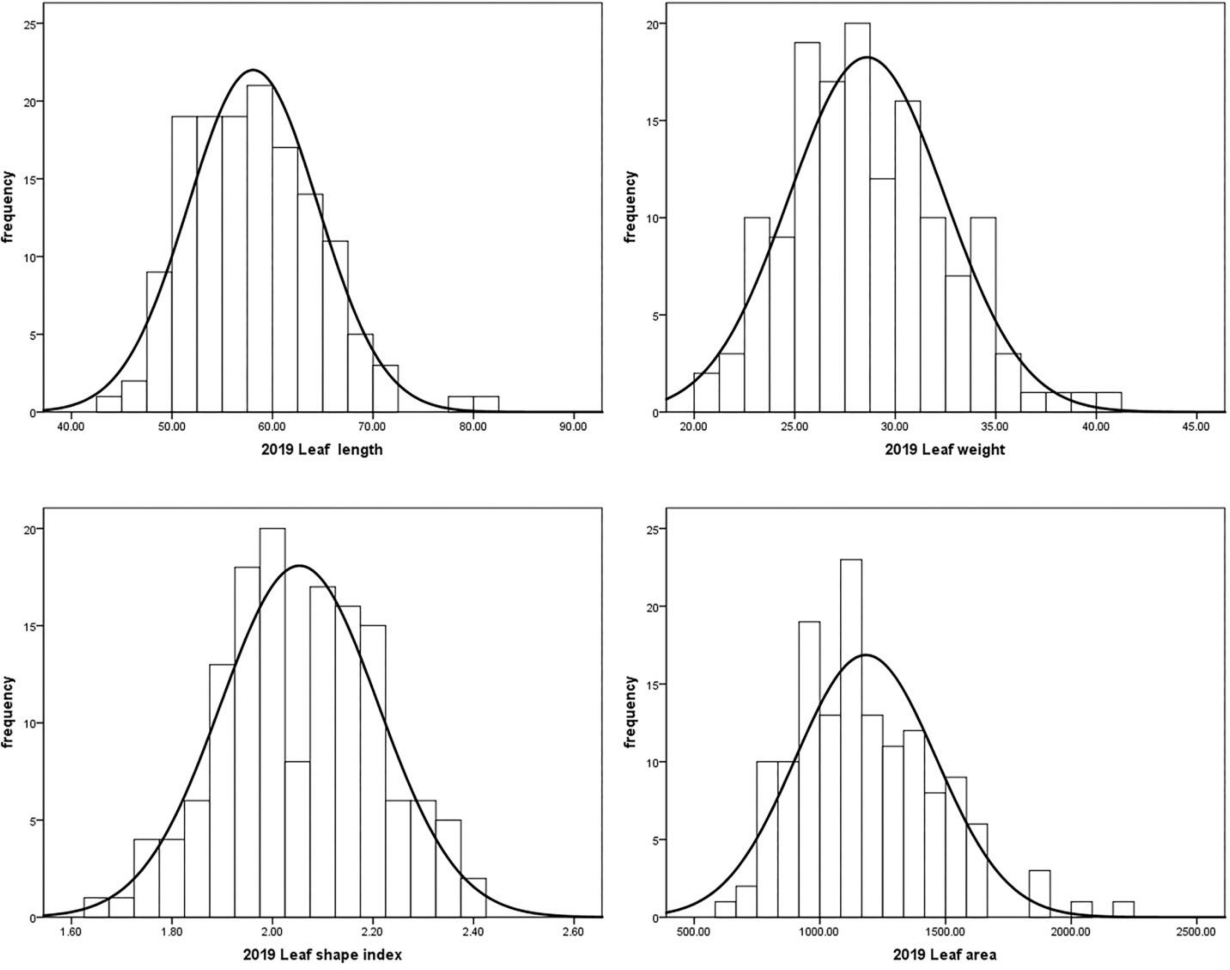
Normal distribution plot of leaf size in 2021.

Leaf traits including leaf length, leaf width, leaf area and leaf shape index of the ‘JMS2’ × ‘Jiaocheng 5’ segregation population were investigated for three years from 2019 to 2021 (**Table 5**). The mean value of leaf length, leaf width, and leaf area of the male parent were all greater than that of the female parent, and the progeny mean value of leaf length, leaf width, and leaf area were all less than the two parents, which showed a trend of small variation. The population mean value of leaf traits varied with the year, and it was speculated that environmental factors could have been responsible for the impacts. In our previous study (Bao et al., 2021), results showed that the leaf area value ranged from 6.31 cm to 22.46 cm in 2019 and from 7.44 cm to 22.46 cm in 2020, and the coefficient of variation (CV) value of leaf area was 22.9% and 27%, while the CV of leaf shape index was 7.67% and 8.05%. After another year of research, we found that the leaf area value ranged from 6.31cm to 35.37cm in 2021, the coefficient of variation (CV) value of leaf area was 29.52%, and the CV of leaf shape index was 9.31% in 2021. The value of leaf traits varied continuously and had a normal distribution according to the Shapiro–Wilk test, demonstrating these traits were typical quantitative traits controlled by polygenes (**Figure 3**).

**Table 5:** The leaf phenotypic traits of mapping population (Bao et al., 2021).

Trait	Year	Male	Female	F1					
				Mean ( ± SD)	Minimum	Maximum	CV (%)	Skewness	Kurtosis
Leaf Length (cm)	2019	8.06	7.00	5.78 ± 0.61	4.45	7.85	10.57	0.36	-0.03
	2020	8.51	6.41	6.31 ± 0.81	4.54	8.98	12.80	0.51	0.47
	2021	8.77	7.74	6.80 ± 1.05	4.15	9.47	11.67	0.34	0.13
Leaf width (cm)	2019	3.78	3.38	2.85 ± 0.38	2.01	4.09	13.35	0.46	0.12
	2020	4.35	3.27	3.15 ± 0.47	2.18	4.66	14.82	0.53	0.07
	2021	4.22	3.70	3.41 ± 0.86	2.01	10.14	23.43	4.94	37.11
Leaf shape index	2019	2.14	2.08	2.05 ± 0.16	1.65	2.41	7.67	-0.02	-0.39
	2020	1.98	1.97	2.02 ± 0.16	1.66	2.47	8.05	0.23	-0.37
	2021	2.13	2.12	2.09 ± 0.20	1.70	2.72	9.31	0.87	0.92
Leaf area (cm^2^)	2019	20.73	16.42	11.73 ± 2.69	6.31	22.46	22.90	0.76	1.02
	2020	25.29	14.24	14.07 ± 3.80	7.44	28.84	27.00	0.95	1.29
	2021	24.66	19.09	15.93 ± 5.3	6.31	35.37	29.52	1.86	6.41

### QTL analysis of leaf traits

**Figure 4:**
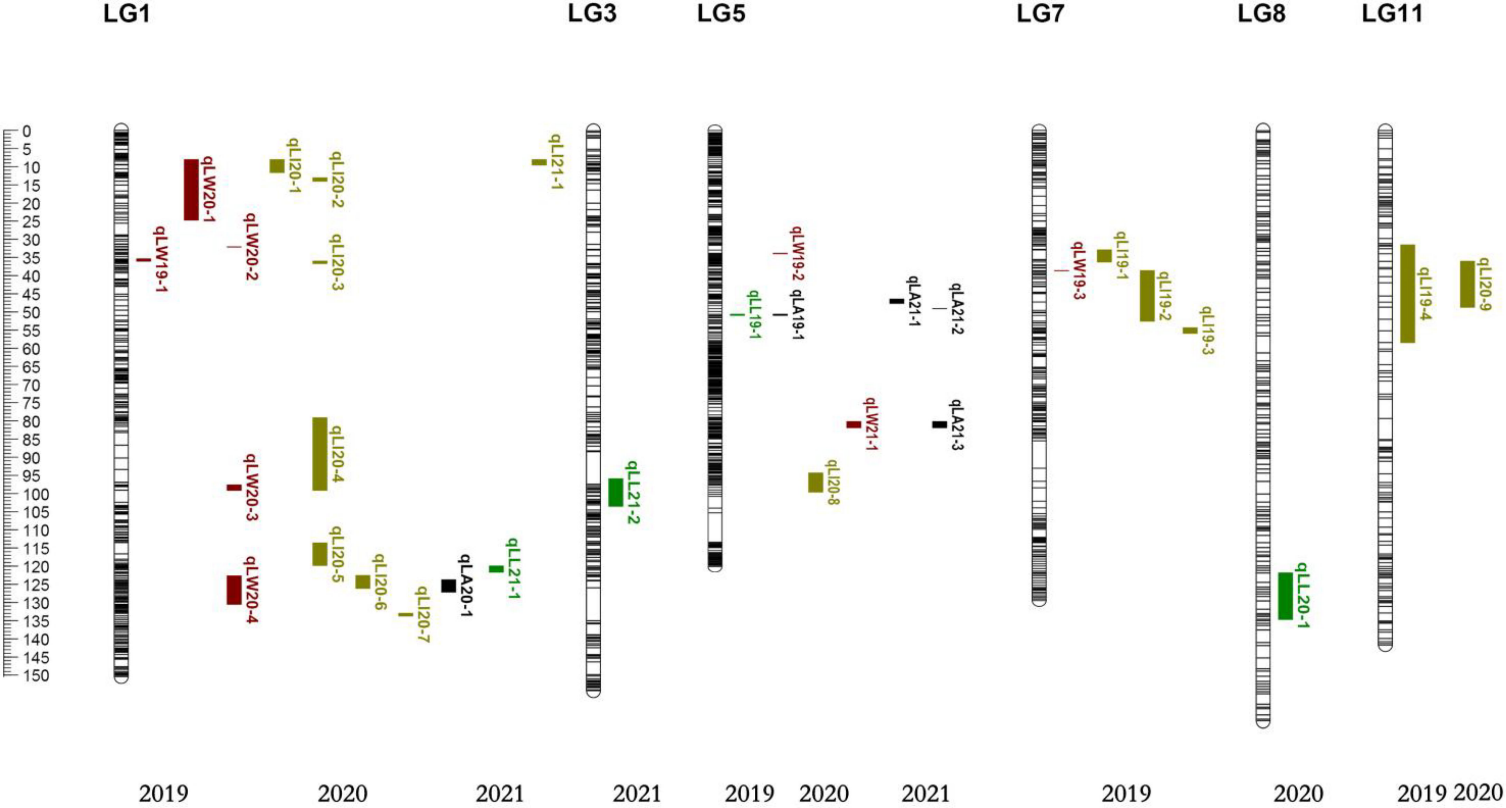
Distribution map of QTL loci for leaf size traits.

**Figure 5:**
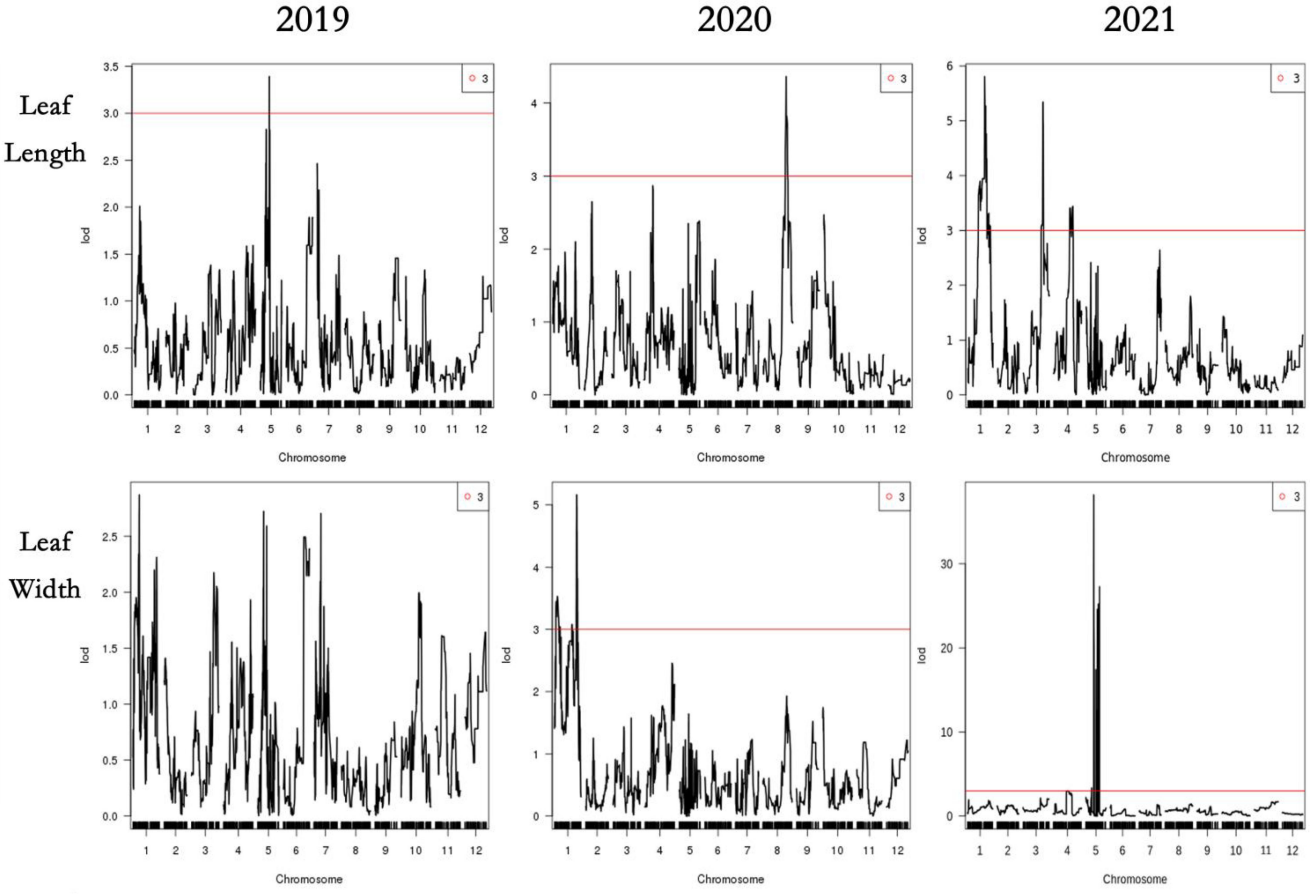
QTL analysis of the 4 leaf traits in 2019,2020 and 2021. The x-axis indicates map position (cM) across the 12 LGs, while the y-axis represents the LOD scores. Horizontal line on the chart represents LOD threshold.

The compound interval mapping method (CIM) was used to ensure the reliability of the identified QTLs in combination with leaf phenotypic traits for three consecutive years. It was regarded as presenting QTL loci when the threshold was greater than 3 (LOD >3), and we generally believed that the major QTLs would exist when the threshold was greater than 3.5 (LOD >3.5). In our study, QTLs were detected by LOD ≥3.0, except for the leaf width QTLs, which were divided by LOD ≥2.7 in 2019. A total of 31 QTLs were detected, with LODs ranging from 2.70 to 27.26 and PVEs from 0.74% to 34.79%, including 17 major QTLs (LOD ≥3.5). There were four-leaf length-related QTLs detected in LG1, LG3, LG5, and LG8, with LODs ranging from 3.3 to 5.34 and PVEs from 3.28% to 14.22%. Eight leave width-related QTLs were located in LG1, LG5, and LG7, with LODs ranging from 2.7 to 27.26 and PVEs from 0.74% to 34.79%, of which the QTL qLW21-1 was detected in LG5 (80.10 cM–81.76 cM) with LOD values of 27.26 and explaining 34.79% of the observed genotypic variation. Fourteen QTLs associated with leaf shape index were detected in LG1, LG5, LG7, and LG11, with LODs ranging from 3.03 to 4.74 and PVEs from 1.42% to 13.85%. In addition, five linked to leaf area QTLs were identified in LG1 and LG5, with LOD values from 3.78 to 21.93 and PVEs from 3.64% to 28.97%, of which the QTL qLA21-3 was detected in LG5 (80.10 cM–81.76 cM) with LOD values of 21.93 and explaining 28.97% of the observed genotypic variation, containing 35 markers (**Table 6**; **Figures 4**, **5**).

**Table 6:** QTLs for leaf traits detected by interval mapping.

Trait	Year	QTL	LG	Intervals on	LOD	Peak	Peak	Exp (%)	Contain markers
				maps (cM)			location (cM)		
Leaf Length (cm)	2019	qLL19-1	5	50.58–50.86	3.39	19564646	50.63	9.16	5
	2020	qLL20-1	8	121.79–134.63	3.82	17584215	129.53	14.22	35
	2021	qLL21-1	1	119.99–121.60	3.30	12830357	120.68	3.28	29
		qLL21-2	3	95.79–103.29	5.34	16743188	116.68	5.09	75
Leaf width(cm)	2019	qLW19-1	1	35.36–36.01	2.87	33054055	36.01	8.48	10
		qLW19-2	5	33.82–33.94	2.72	19615406	33.82	6.26	5
		qLW19-3	7	38.60–38.60	2.70	19596052	38.6	6.05	8
	2020	qLW20-1	1	8.05–24.74	3.53	35127889	16.74	2.48	55
		qLW20-2	1	32.10–32.10	3.04	33996476	32.1	1.66	2
		qLW20-3	1	97.71–99.13	3.08	24069819	99.13	0.74	13
		qLW20-4	1	122.71–130.45	5.16	10609837	124.68	5.69	112
	2021	qLW21-1	5	80.10–81.76	27.26	11985371	81.67	34.79	36
Leaf shape index	2019	qLI19-1	7	32.85–36.17	3.47	20427573	35.48	2.17	11
		qLI19-2	7	38.60–52.52	4.74	17699985	44.69	6.87	57
		qLI19-3	7	54.25–55.82	3.61	16606352	55.59	3.47	52
		qLI19-4	11	31.47–58.32	3.69	13689944	35.98	11.05	30
	2020	qLI20-1	1	8.05–11.58	3.79	35346999	9.53	3.6	25
		qLI20-2	1	13.12–13.99	3.04	35288121	13.12	2.46	2
		qLI20-3	1	36.01–36.67	3.45	33053790	36.23	3.43	12
		qLI20-4	1	79.24–99.13	3.96	24072860	83.25	1.42	59
		qLI20-5	1	113.55–119.79	3.25	11639788	119.19	4.03	25
		qLI20-6	1	122.57–126.09	3.77	11034401	124.97	2.8	61
		qLI20-7	1	132.98–133.69	3.03	7951976	133.69	1.83	6
		qLI20-8	5	94.21–99.49	3.99	8634144	94.33	6.58	130
		qLI20-9	11	35.98–48.62	3.22	13689944	35.98	4.91	13
	2021	qLI21-1	1	8.05–9.52	3.24	35404114	9.53	13.85	18
Leaf area (cm^2^)	2019	qLA19-1	5	50.58–50.86	4.12	19564652	50.63	11.78	5
	2020	qLA20-1	1	123.84–127.07	3.78	9952647	125.25	12.35	71
	2021	qLA21-1	5	46.41–47.62	18.33	19559405	47.11	3.64	5
		qLA21-2	5	48.98–48.98	14.76	19556502	48.98	4.91	1
		qLA21-3	5	80.10–81.76	21.93	11985371	81.67	28.97	35

### Quantitative trait loci clusters and candidate gene prediction

There were six overlapping intervals among the QTL loci for leaf size traits (**Table 7**). The QTLs associated with the leaf shape index were identified corresponding to their genetic distance intervals of 31.47 cM–58.32 cM and 35.98 cM–48.62 cM on LG11, with a LOD score of 3.69 and 3.22, explaining 11.05% and 4.91% of phenotypic variation in 2019 and 2020, respectively. The location of 50.58 cM–50.86 cM mapped on LG5, with a LOD score of 3.39 and 4.12, explained 9.16% and 11.78% phenotypic variation of leaf length and leaf area in 2019, respectively. The location of 80.10 cM–81.76 cM mapped on LG5, with a LOD score of 27.26 and 21.93, explained 34.79% and 28.97% of the phenotypic variation of leaf width and leaf area in 2021. There were 3 overlaps (qLW20-4, qLI20-6, and qLA20-1) with distance intervals of 123.84 cM–126.09 cM on LG1, which were linked to leaf width, leaf shape index, and leaf area simultaneously. There were other 3 overlaps (qLW20-1, qLI20-1, and qLI21-1) with distance intervals of 8.05 cM–9.52 cM on LG1, which were linked to leaf width, leaf shape index, and leaf area simultaneously. One overlapping interval (qLW20-1, qLI20-2) between leaf width and leaf shape index was identified with distance intervals of 13.12 cM–13.99 cM on LG1. These genetic intervals identified with stable QTL effects deserve special attention in follow-up studies.

**Table 7:** QTL cluster information of leaf size traits.

Traits	Correspond QTL	Coincidence interval (cM)	Marker number	LG
Leaf width, Leaf shape index	qLW20-1,qLI20-1, qLI21-1	8.05–9.52	18	1
Leaf width, Leaf shape index	qLW20-1, qLI20-2	13.12–13.99	2	1
Leaf width, Leaf shape index, Leaf area	qLW20-4,qLI20-6,qLA20-1	123.84–126.09	56	1
Leaf Length,Leaf area	qLL19-1, qLA19-1	50.58–50.86	5	5
Leaf width, Leaf area	qLW21-1, qLA21-3	80.10–81.76	36	5
Leaf shape index	qLI19-4, qLI20-9	35.98–48.62	13	11

To delve deeper into the markers and genes associated with leaf phenotypic traits, we compared the KEGG and GO databases to determine the tag information for the associated region and the markers as well as genes in the associated regions (**Table 8**). A total of 16,355 and 1,769 genes with functional SNP and InDel markers, respectively, as well as 2,829 genes with functional variation were compared. The KEGG and GO databases were compared to 724 and 2,982 genes, respectively. According to the physical positions of overlapping QTL fragments, three candidate regions for leaf size traits were identified, suitable candidate genes were directly searched for in the relevant regions, and their functions were predicted (**Table 9**). There were two QTL clusters in LG1. Five candidate genes in the genomic region were obtained from the reference genome database, and three candidate genes were screened in LG5. These candidate genes related to leaf size were involved in cell wall biosynthesis (LOC107433386, LOC107417968, LOC112492570, LOC112491602, LOC107418569, and LOC107418597) and cell division (LOC107418196 and LOC107418241).

**Table 8:** Markers and genes in the associated regions by comparing the data bases.

LG	Coincidence interval (cM)	QTL loci	Gene number	Gene withEff variation	KEGG	GO comments	Eff SNP	Eff InDel
					comments			
1	8.05–9.52	qLW20-1	390	239	69	223	1127	146
		qLI20-1	378	239	69	223	1076	134
		qLI21-1	363	235	97	331	994	123
1	13.12–13.99	qLI20-2	27	4	1	4	133	23
1	123.84–126.09	qLW20-4	309	177	28	168	1573	155
		qLI20-6	263	147	23	140	1224	126
		qLA20-1	249	135	18	130	1165	126
5	50.58–50.86	qLL19-1	0	0	0	0	0	0
		qLA19-1	0	0	0	0	0	0
5	80.10–81.76	qLW21-1	91	62	6	83	498	44
		qLA21-3	91	62	6	83	498	44
11	35.98–48.62	qLI19-4	209	104	21	96	601	42
		qLI20-9	20	11	1	8	208	21

**Table 9:** Quantitative trait loci clusters and candidate gene functional predictions.

QTL clusters	Range (cM)	QTL name	Candidate genes	Function annotation
1	8.05–9.52	qLW20-1 qLI20-1 qLI21-1	LOC107433386	receptor-like protein 6
1	123.84–126.09	qLW20-4 qLI20-6 qLA20-1	LOC107417968 LOC112492570 LOC107418196 LOC107418241	wall-associated receptor kinase 2-like wall-associated receptor kinase 3-like AMN1 and F-box protein F-box protein At2g26160-like
5	80.10–81.76	qLW21-1 qLA21-3	LOC112491602 LOC107418569 LOC107418597	probable LRR receptor-like serine/threonine-protein kinase At3g47570 probable LRR receptor-like serine/threonine-protein kinase At3g47570 probable beta-D-xylosidase 5

## Discussion

### Construction of the genetic map

The high-saturate molecular linkage map is an important tool for elucidating the genetic basis for key traits of interest and MAS-based breeding, which may help in the fine mapping of quantitative trait loci (QTL). However, there were obvious problems, including the inconsistency between LG number and chromosome number and large spacing among markers in the previous genetic linkage map in Chinese jujube constructed by AFLP, RAPD, and SSR, which affected the accuracy of QTL (Lu, 2003; Shen, 2005; Qi, 2006; Xu, 2012). So far, some of the highest density genetic linkage maps of jujube have been constructed over the years. For example, the genetic linkage map of ‘JMS2’ × ‘Xing 16’ was published based on the RAD-Tag method and contained a total of 2,748 SNP markers spanning 913.87 cM, with an average distance of 0.34 cM between adjacent markers (Zhao et al., 2014). The genetic map of ‘Dongzao’ × ‘Yingshanhong’ constructed by restriction-site-associated DNA sequencing (RAD-seq) consisted of 4,669 markers (4,137 SNPs, 486 InDel, and 46 SSRs) and spanned 2,643.79 cM with an average marker interval of 0.57 cM (Zhang et al., 2016).The genetic map of ‘Dongzao’ × ‘Zhongningyuanzao’ was constructed by sequencing strategy (GBS), which spanned 1,456.53 cM and contained 2,540 SNP markers with an average marker interval of 0.88 cM (Zhang et al., 2016).The most recent jujube genetic map of ‘Dongzao’ × ‘Jinsi 4’ was produced by GBS strategy and developed a total of 3,792 SNP markers spanning 2,167.5 cM, with an average marker interval distance of 0.358 cM (Wang et al., 2019). In this study, Illumina sequencing approaches were employed to facilitate the re-sequencing of 140 F1 jujube progeny and their parents, yielding 203.02 GB of raw data from which a high-density genetic map was constructed by the WGR method. Relative to other previously reported genetic linkage maps, our map is superior with a marker number of 8,684 (including 8,158 SNP and 526 InDel) and a smaller marker spacing of 0.20 cM. The LG5 presented the most markers (1,684 SNP and 108 InDel) and the smallest average distance between the markers of 0.07 cM and the shortest total genetic distance of 120.03 cM in the integrated maps. Only 17 of ‘Gap >5’ were observed and the LG1, LG4, and LG8 had no ‘Gap >5,’ which indicated good uniform coverage and highly saturated linkage maps. The number of markers in our map was roughly 2-fold higher than the reported high-density map (Zhang et al., 2016; Wang et al., 2019), and such increased density enables more accurate QTL detection and can better facilitate potential candidate genes associated with key plant traits. However, the total distance of this map was 1,713.22 cM, which was shorter than the spanned 2,643.79 cM reported by Zhang et al. (2016), which may be caused by differences in hybrid population materials, sequencing methods, mapping software, etc. It could be found that the total length of the genetic map was not proportional to the average spacing between markers in apple (MD Clark et al., 2014; Gardner et al., 2014) and watermelon (Nimmakayala et al., 2014; Zhang et al., 2018). This map can serve as a high-quality reference to support candidate gene identification, molecular breeding, map-based gene cloning, and marker-assisted selection of jujube.

### Quantitative trait locus identification

The leaf-related traits such as area and size determine the photosynthetic capacity of plants, thereby these traits regulate potential fruit yield and quality (Zhang et al., 2021). Thus, the genetic factors responsible for the regulation of leaf traits in jujube trees are invaluable for both selective breeding efforts and clarification of the mechanisms governing plant developmental biology. The construction of an F1 population and mapping of QTLs in jujube is challenging because jujube is a self-incompatible plant with a small flower size and a high seedless percentage, so it is difficult to generate an F1 population by traditional crossbreeding (Wang et al., 2019). With the development of cross-breeding by controlled honeybee-assisted pollination by nets in jujube, a genetic mapping population of ‘JMS2’ and ‘Jiaocheng 5’ was successfully constructed, which provided the opportunity for QTL analysis for leaf traits. A LOD value is a reflection of the recombination frequency or linkage distance between predicted genes and traits (Khan et al., 2013). The LOD threshold of 2.5 was used to identify potential QTLs, and a significant LOD threshold was calculated by permutation test as 3.5 at the 95% confidence level, which can effectively control the occurrence of false positive loci (Zhang et al., 2012; Wu et al., 2014). Previous studies on QTL mapping of leaf phenotypical traits in jujube have rarely been precise sections on the linkage group. For example, Shen (2005) first detected 25 QTLs for leaf traits using AFLP markers, but the genetic maps constructed were of lower resolution and unsaturated, and possibly unable to reliably capture QTLs. Wang et al. (2019) detected four QTLs for leaf length on LG1 and LG11, five QTLs for leaf width on LG1 and LG11, four QTLs for leaf area on LG9 and LG11, and seven QTLs for leaf shape index on LG1 and LG11. In this study, a total of 31 QTLs, including 17 major QTLs, were detected, of which four QTLs for leaf length on LG1, LG3, LG5, and LG8, eight QTLs for leaf width on LG1, LG5, and LG7, 14 QTLs for leaf shape index on LG1, LG5, LG7, and LG11, and five QTLs for leaf area were identified on LG1 and LG5. Significantly, the qLW21-1 for leaf width and qLA21-3 for leaf area were detected simultaneously in LG5 (80.10 cM–81.76 cM) with LOD values of 27.26 and 21.93, explaining 34.79% and 28.97% of the observed genotypic variation, respectively. In addition, the QTLs associated with leaf length and leaf area were located in the same linkage group (LG1, LG5, and LG11) as those in previous studies, although at different positions (Shen, 2005; Wang et al., 2019).

QTLs and genes can exhibit pleiotropic effects on multiple traits, and phenotypically correlated traits are often mapped together (Jia et al., 2016; Wang et al., 2019). Most of the evaluated traits showed significant differences between years, suggesting that environmental orchard conditions in an orchard affect phenotypic characters (Zhang et al., 2022). Hence, the collection of reliable phenotypic data and continuous trait localization for many years is a critical step in the identification of genomic markers. Quantitative trait loci clusters are regions of the chromosome containing multiple loci associated with a range of traits (Kochevenko et al., 2018; Guan et al., 2020).

In this study, six overlapping intervals among the QTL loci for leaf size traits were clearly observed in some chromosomal intervals. Three QTL clusters were detected on LG1. The overlaps of 123.84 cM–126.09 cM included three QTLs (qLW20-4, qLI20-6, and qLA20-1) with LOD threshold higher than 3.5, which were related to leaf width, leaf shape index, and leaf area simultaneously. The overlaps of 8.05 cM–11.58 cM included three QTLs (qLW20-1, qLI20-1, and qLI21-1) with LOD threshold higher than 3.5, which were related to leaf width and leaf shape index and leaf area simultaneously, suggesting that there were major genes affecting leaf phenotypic traits in this region. The overlaps of 13.12 cM–13.99 cM included two QTLs (qLW20-1, qLI20-2) were related to leaf width and leaf shape index. It was found that LG1 had the most QTL loci related to leaf phenotypic traits, including leaf width, leaf area, and leaf shape index. Two QTL clusters were detected on LG5, including the location of 50.58 cM–50.86 cM with a LOD score of 3.39 and 4.12, explaining 9.16% and 11.78% phenotypic variation of leaf length and leaf area in 2019, respectively, and the location of 80.10 cM–81.76 cM with a LOD score of 27.26 and 21.93, explaining 34.79% and 28.97% phenotypic variation of leaf width and leaf area in 2021. It was speculated that there were stable genes affecting the leaf area in this region. In addition, one QTL cluster were detected on LG11 with genetic distance intervals of 31.47 cM–48.62 cM, with a LOD score of 3.69 and 3.22, explaining 11.05% and 4.91% of phenotypic variation in 2019 and 2020, respectively. Such clusters are of particular relevance to breeders, as they can thereby focus their efforts on QTL regions associated with the greatest degree of phenotypic variance. These newly identified QTL clusters would be valuable resources for further screening and verification of candidate genes related to leaf traits in jujube.

### Candidate gene screening and analysis

In the current genetic study, candidate gene searches based on QTL intervals are common. Within these three cluster ranges, eight potentially relevant genes were identified. The genes with functional variation were analyzed by comparing KEGG and GO databases to further clarify the functions of genes within QTLs. It is generally known that leaf size traits are determined by the coordination of cell proliferation and cell expansion during leaf development (Gonzalez et al., 2012). Two genes (LOC107418196, LOC107418241) are predicted to encode ‘AMN1 and F-box protein’ and ‘F-box protein At2g26160-like,’ which is a key regulator of leaf size by regulating plant cell division and the number of cells. This trend has also been observed in other species. For example, the difference in the size of leaves of *Populus deltoides* ‘Danhong’ and *Populus simonii* ‘Tongliao1’ was related to the number of cells and is affected by cell division and chromosome duplication (Zhang et al., 2021a). Zhou et al. (2021) showed that MINI ORGAN1 (MIO1) encodes the F-Box protein, which increases leaf cell proliferation by regulating cell division during the growth and development of leaves. These two candidate genes represent ideal targets for future cloning and functional verification studies.

In addition, the other two genes in this cluster include LOC107417968 and LOC112492570, which encode ‘wall-associated receptor kinase 2-like’ and ‘wall-associated receptor kinase 3-like, ‘ expressed cell wall-associated kinases (WAKs) and play a crucial role in maintaining cell wall integrity during plant growth (Jose et al., 2020). It was found that receptor-like kinases (RLKs) and receptor-like proteins (RLPs) are the main cell surface receptors of plants. Among them, receptor-like kinases (RLKs) are protein kinases located on the cell membrane and play an important role in the process of plant growth and development. The extrinsic information is efficiently transmitted into the nucleus of the plant cell. Although receptor-like proteins (RLP) are structurally distinct from receptor-like kinases, their proteins are similar (Jose et al., 2020; Wang and Gou, 2020; Li et al., 2021).

In this study, the other four receptor genes (LOC107433386, LOC112491602, LOC107418569, and LOC107418597) are annotated as ‘receptor-like protein 6,’ ‘probable LRR receptor-like serine/threonine-protein kinase At3g47570,’ and ‘probable beta-D-xylosidase 5,’ which might play an important role in plant growth and development. Wu et al. (2017) found that leucine-rich repeat receptor-like kinases (LRR-RLKs) exist in different plants and are highly representative. LRR-RLKs have the effect of promoting cell expansion. At the same time, several genes related to cell wall biosynthesis were identified, such as beta-D-xylosidase 5, whose high enzymatic activity is likely to be related to the necessary modification of the cell wall, thereby affecting cell size and ultimately determining leaf size. (Minic et al., 2004; Chavez Montes et al., 2008). The specific roles of these genes will help to elucidate the regulatory mechanisms of jujube leaf phenotypic traits and be a focus of future research efforts.

## Conclusions

A highly saturated genetic map of jujube with the most markers and the smallest average distance up to now was constructed using the whole genome resequencing method and an F1 segregation population of 140 individuals derived from a cross between ‘JMS2’ × ‘Jiaocheng 5’. A total of 8,684 markers, including 8,158 SNP and 526 InDel markers, were distributed in all the 12 linkage groups, spanning 1,713.22 cM with an average marker interval of 0.2 cM. Among them, the LG5 contained the largest number of markers (1,684 SNP and 108 InDel). Based on the genetic map and the phenotype data, a total of 31 leaf trait QTLs, including 17 major QTLs, were identified, with 4, 8, 14, and 5 QTLs contributing to leaf length, leaf width, leaf shape index, and leaf area, respectively. Six QTLs clusters were detected on LG1 (8.05 cM–9.52 cM; 13.12 cM–13.99 cM; 123.84 cM–126.09 cM), LG5 (50.58 cM–50.86 cM; 80.10 cM–81.76 cM), and LG11 (35.98 cM–48.62 cM). Eight candidate genes were identified within the QTL cluster regions. Annotation analysis showed that four genes (LOC107418196, LOC107418241, LOC107417968, and LOC112492570) in these QTLs are related to cell division and cell wall integrity. This research will provide a valuable tool for further QTL analysis, candidate gene identification, map-based gene cloning, comparative mapping, and marker-assisted selection (MAS) in jujube.

## Data availability statement

The data presented in the study are deposited in the NCBI repository, accession number is PRJNA877294 (https://www.ncbi.nlm.nih.gov/bioproject/PRJNA877294).

## Author contributions

FY and CW conceived and designed the experiments. YL and JB performed the experiments. FY, YL, and JB analyzed the data and wrote the manuscript. YP, JW, and ML participated in the experiments and analysis. CW, JW, and ML edited the manuscript. All authors contributed to the article and approved the submitted version.

## Funding

This work was supported by the National Science Foundation of China (32060656), the Major Scientific and Technological Projects of XPCC (2017DB006), the Open Fund Project of Key Laboratory of Biological Resources Protection and Utilization of Xinjiang Production and Construction Corps in Tarim Basin (BRZD1811), and the Hebei Province Key R&D Program (20326811D). Innovation and Entrepreneurship Platform and Base Construction Project of XPCC (2019CB001).

## Acknowledgments

We thank Dr. Noor Muhammad from Pakistan and Dr. Lixin Wang, who got his PhD from KIT University, Germany for their kind polishing of the manuscript.

## Conflict of interest

The authors declare that the research was conducted in the absence of any commercial or financial relationships that could be construed as a potential conflict of interest.

## Publisher’s note

All claims expressed in this article are solely those of the authors and do not necessarily represent those of their affiliated organizations, or those of the publisher, the editors and the reviewers. Any product that may be evaluated in this article, or claim that may be made by its manufacturer, is not guaranteed or endorsed by the publisher.
